# IL28B SNP rs8099917 Is Strongly Associated with Pegylated Interferon-α and Ribavirin Therapy Treatment Failure in HCV/HIV-1 Coinfected Patients

**DOI:** 10.1371/journal.pone.0013771

**Published:** 2010-10-29

**Authors:** Ester Aparicio, Mariona Parera, Sandra Franco, Nuria Pérez-Alvarez, Cristina Tural, Bonaventura Clotet, Miguel Angel Martínez

**Affiliations:** 1 Fundació irsiCaixa, Hospital Universitari Germans Trias i Pujol, Universitat Autonoma de Barcelona (UAB), Badalona, Barcelona, Spain; 2 Fundació de la Lluita contra la Sida, Hospital Universitari Germans Trias i Pujol, Badalona, Spain; 3 Statistics and Operations Research Department, Technical University of Catalonia, Barcelona, Spain; University of Sao Paulo, Brazil

## Abstract

Recent genome-wide association studies report that the SNP rs8099917, located 8.9 kb upstream of the start codon of IL28B, is associated with both disease chronicity and therapeutic response to pegIFN-α and RBV in patients infected with genotype 1 HCV. To determine the effect of rs8099917 variation on the response of HCV to therapy, we genotyped this variant in a cohort of 160 HCV/HIV-1 coinfected patients in our clinic unit who received combined peg-IFN-α/RBV therapy. The rs8099917 T/G or G/G genotypes were observed in 56 patients (35%). Treatment failure occurred in 80% of G-allele carriers versus 48% of non-carriers (P<0.0001). This result reveals that the G allele was strongly associated with treatment failure in this patient cohort. Importantly, a highly significant association was found between the G-allele and response to therapy in HCV genotype 1-infected patients (P<0.0001) but not in HCV genotype 3-infected patients. Multivariate analysis (odds ratio; 95% confidence interval; P value) indicated that the rs8099917 TT genotype was a strong predictor of treatment success (5.83; 1.26–26.92; P = 0.021), independent of baseline plasma HCV-RNA load less than 500 000 IU/ml (4.85; 1.18–19.95; P = 0.025) and absence of advanced liver fibrosis (5.24; 1.20–22.91; P = 0.025). These results reveal the high prevalence of the rs8099917 G allele in HCV/HIV-1 coinfected patients as well as its strong association with treatment failure in HCV genotype 1-infected patients. rs8099917 SNP genotyping may be a valid pre-treatment predictor of which patients are likely to respond to treatment in this group of difficult-to-treat HCV/HIV-infected patients.

## Introduction

The hepatitis C virus (HCV), a positive-stranded RNA virus, is the causal agent of chronic liver infection afflicting more than 170 million people worldwide [1]. HCV infection is usually persistent, with 70–80% of patients becoming chronic carriers. After an asymptomatic period that often lasts for years, many patients develop chronic liver disease, including cirrhosis and hepatocellular carcinoma [2].

The standard of care for patients with chronic hepatitis C is pegylated interferon alpha (peg-IFN-α) in combination with ribavirin (RBV) [3]. A positive response to treatment is defined as a sustained virological response (SVR; a negative hepatitis C PCR test 6 months after cessation of therapy). The SVR rate for individuals infected with HCV genotypes 1 or 4 ranges between 40 and 50% and requires 12 months of therapy. Patients infected with HCV genotypes 2 or 3 typically achieve SVRs of nearly 75% after only 6 months of therapy [4], [5], [6], [7]. HCV genotype is the most important predictive factor for the treatment response of patients with chronic hepatitis C; however, host factors such as age, sex, race, liver fibrosis, and obesity have also been associated with peg-IFN-α/RBV therapy outcome [8], [9].

Chronic HCV infection in human immunodeficiency virus type 1- (HIV-1-) positive patients is a frequent and emerging health problem. HCV and HIV-1 are both transmitted by blood and blood products. Coinfection is therefore common in people with high exposure to blood. Hepatitis C is found in 60 to 90% of HIV-1-positive hemophiliacs and in 50 to 70% of HIV-1-positive intravenous drug users [10]. Although sexual transmission of HCV is rare, small epidemics of acute hepatitis C have been reported recently in homosexual men. In our clinic unit, nearly 50% of the HIV-1 patients are coinfected with HCV [11]. In the highly active antiretroviral therapy (HAART) era, HCV/HIV-1 coinfection increases the risk of hospitalization and death compared with HIV-1 infection alone [12]. Importantly, standard therapy with peg-IFN-α/RBV elicits significantly lower rates of SVR in HCV/HIV-1 coinfected patients than in HCV monoinfected individuals. Among patients infected with HCV genotypes 1 or 4, the SVR rate is only about 30%. The corresponding rate among patients infected with HCV genotype 2 or 3 is closer to 60% [13], [14]. These results illustrate the difficulties in successfully treating HCV infection in HCV/HIV-1 coinfected patients.

Four genome-wide association studies recently reported associations of several single nucleotide polymorphism (SNP) in the IL28B gene on chromosome 19, which encodes type III IFN-λ, with the response to HCV peg-IFN-α/RBV-based therapy [15], [16], [17], [18]. Interestingly, the rs8099917 SNP was the only SNP in these four studies that was strongly associated with response to therapy, but the studies did not specifically investigate the association between IL28B variation and response to peg-IFN-α/RBV-based therapy in HCV/HIV-1 coinfected patients. Recently, the rs12979860 SNP, also located near the IL28B gene, has been associated with HCV treatment response in HIV-1-infected patients with chronic hepatitis C due to genotypes 1 or 4 [19], [20]. To directly address the role of the rs8099917 SNP in HCV treatment response in HCV/HIV-1 coinfected patients, we genotyped 160 individuals from our clinic unit who were treated with peg-IFN-α/RBV combination therapy.

## Results

### rs8099917 SNP prevalence

Of the 160 patients in the study, 86 (54%) were chronically infected with HCV genotypes 1, 46 (29%) with HCV genotype 3, and 28 (17%) with HCV genotype 4 (Table 1). To genotype the rs8099917 SNP, we developed a direct sequencing PCR-based protocol. Genotyping revealed a high prevalence of G alleles (TG or GG) in our study cohort (n = 56, 35%) (Table 2). rs8099917 genotypes were in the Hardy-Weinberg equilibrium (P = 0.118). No significant associations were found between the rs8099917 SNP genotype and patient sex, age, CD4+ T cell count, HBV infection, liver enzyme levels, liver fibrosis, or HCV RNA viral load (data not shown). The proportion of the rs8099917 TT, TG, and GG genotypes were 60%, 35%, and 5% among HCV genotype 1 patients; 78%, 15%, and 7% among genotype 3 patients; and 57%, 29%, and 14% among genotype 4 patients. Genotypes 1 and 4 showed similar proportion of G alleles, 40% and 43%, respectively. Interestingly, there was a significant difference between the prevalence of G alleles in HCV genotype 3-infected patients, 22%, and the prevalence in patients infected with HCV genotypes 1 or 4 (P<0.05, Chi-square test). The lower proportion of the G allele in genotype 3-infected patients suggested that G carriers may be less prone to be chronically infected by genotype 3 viruses or, alternatively, that the rs8099917 homozygous TT genotype is not associated with spontaneous clearance of HCV genotype 3.

**Table 1:** Clinical Characteristics of patients with chronic HIV-1 and HCV co-infection and treated with peg-IFN-α/RBV therapy.

		Treatment Success	Treatment Failure	P
Patients, n (%)		67 (42%)	93 (58%)	-
Age (mean ± SEM)		48.42±0.6773	47.21±0.6283	ns
Gender, n (%)				ns
	Female	24 (45%)	29 (55%)	
	Male	43 (40%)	64 (60%)	
CD4+ (cell counts/mL) (mean ± SEM)		597.9±27.82	584.6±31.85	ns
HCV genotype n (%)				<0.005
	1	29 (34%)	57 (66%)	
	3	29 (63%)	17 (37%)	
	4	9 (32%)	19 (68%)	
Fibrosis stage n (%)				<0.05
	0–1	12 (36%)	21 (64%)	
	2	3 (18%)	14 (82%)	
	3	1 (11%)	8 (89%)	
	4	2 (9%)	21 (91%)	
ALT (U/L) (mean ± SEM)		90.84±10.90	85.54±5.039	ns
AST (U/L) (mean ± SEM)		60.31±6.522	62.35±3.248	ns
HCV RNA (IU/mL) (mean ± SEM)		5.808±0.097	6.036±0.050	<0.05
Undetectable HIV-1 RNA, n (%)		55 (82%)	75 (81%)	ns

Age, Mann-Whitney U test; Gender, Chi-square test; CD4+T cell count, Mann-Whitney U test; HCV genotype Chi-square test; Fibrosis stage, Chi-square test between F0-2 and F3-4, fibrosis data was missing in 78 patients; ALT and AST, Mann-Whitney U test; HCV RNA, unpaired T test; Undetectable HIV-1, Chi-square test.

**Table 2:** Association of rs8099917 SNP with response to peg-IFNα/RBV treatment.

	Treatment Success (SVR) n = 67 (42%)		Treatment Failure n = 93 (58%)		
HCV genotype	TT	TG or GG	TT	TG or GG	P value
1n = 86 (54%)	26 (90%)	3 (10%)	26 (46%)	31 (54%)	<0.0001
3n = 46 (29%)	23 (79%)	6 (21%)	13 (76%)	4 (24%)	ns
4n = 28 (17%)	7 (78%)	2 (22%)	9 (47%)	10 (53%)	ns

To calculate P values (Chi-square test), patients were stratified in two groups according to host polymorphism (host rs8099917 G allele carriers (TG and GG) versus noncarriers (TT). SVR, sustained virologic response; ns, not significant, to calculate.

### rs8099917 SNP and treatment response

The clinical characteristics and HCV treatment responses of the 160 HCV/HIV-1 coinfected patients included in the study are summarized in Table 1. Most patients were on antiretroviral therapy (159, 99.5%), and most patients had controlled HIV-1 replication (130, 81%). Sixty-seven patients (42%) responded successfully to HCV treatment (i.e. achieved SVR). The proportion of patients infected with HCV genotype 3 who achieved SVR (63%) was significantly higher than in patients infected with HCV genotypes 1 or 4 (34% and 32%, respectively) (P<0.05, Chi-square test). A significantly higher HCV RNA viral load was observed in patients who failed treatment (P<0.05, unpaired t test). Likewise, severe fibrosis was significantly associated with treatment failure (P<0.05, Chi-square test) (Table 1). These three clinical parameters, HCV genotype, HCV RNA viral load, and severe fibrosis, are known to be associated with treatment response. We found no significant differences in our study cohort between those who achieved SVR and those who did not with respect to sex, age, HIV-1 viral load, liver enzymes, or CD4+ T cell count (Table 1).

**Figure 1:**
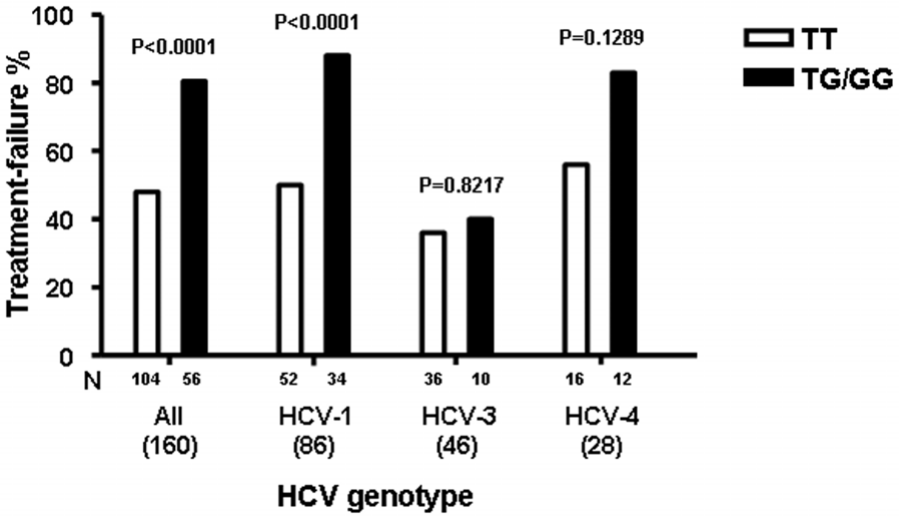
Rate of treatment failure according to rs8099917 SNP genotype in patients with hepatitis C virus (HCV) genotype 1, 3, and 4. P values correspond to Chi-square test.

We then correlated the rs8099917 SNP genotype with the HCV peg-IFN-α/RBV treatment response. The proportions of rs8099917 TT, TG, and GG genotypes were 51.5%, 41.0%, and 7.5% among patients with treatment failure, versus 83.5%, 10.5% and 6% among those with SVR. Overall, allele G carriers had a significantly higher risk of treatment failure than patients carrying the TT genotype (P<0.0001, Chi-square test). This suggests that this rs8099917 SNP may predict treatment failure before peg-IFN-α/RBV therapy. When the data was stratified by HCV genotype, we found that of patients infected with HCV genotype 1, 91% of the rs8099917 G allele carriers had treatment failure (Fig. 1). Among patients infected with HCV genotype 1, the association between the rs8099917 G allele and treatment failure was highly significant (P<0.0001, Chi-square test) (Table 2). Similarly, of patients infected with HCV genotype 4, 83% of the rs8099917 G allele carriers failed treatment (Fig. 1 and Table 2). The sample size of patients infected with HCV genotype 4 was small (n = 28), limiting our ability to detect a significant association between the G allele and treatment failure. In contrast to patients infected with HCV genotypes 1 and 4, HCV genotype 3-infected patients that failed therapy had a similar proportion of TT and TG/GG genotypes (36% and 40%, respectively) (Fig. 1). Thus for patients infected with HCV genotype 3, there was no significant association between rs8099917 SNP genotype and treatment failure (Table 2).

Next, factors associated with treatment failure as HCV genotype (P<0.005), fibrosis stage (P<0.05), higher pretreatment HCV RNA viral load (P<0.05) and rs8099917 SNP genotype (P<0.0001) (Table 1) were subjected to univariate and multivariate logistic regression analysis (odds ratio; 95% confidence interval; P value). In the univariate analyses, rs8099917 SNP genotype TT (4.73; 2.19–10.20; P = 0.00006), baseline HCV- RNA viral load less than 500 000 IU/ml (3.10; 1.44–6.66; P = 0.003), absence of severe liver fibrosis (stages F0–F2) (4.14; 1.06–16.04; P = 0.036) and HCV genotype 3 (3.20; 1.58–6.49; P = 0.001) were significantly associated with treatment success. When a multivariate model was built with the previous factors, the rs8099917 TT genotype remained a strong predictor of treatment success (5.83; 1.26–26.92; P = 0.021), independently of baseline plasma HCV-RNA load less than 500 000 IU/ml (4.85; 1.18–19.95; P = 0.025) and fibrosis stages F0–F2 (5.24; 1.20–22.91; P = 0.025).

## Discussion

HIV-1 accelerates the course of HCV-associated liver disease. SVR after peg-IFN-α/RBV therapy in patients coinfected with HCV/HIV-1 reduces liver-related complications and mortality [22]. However, side effect rates for anti-HCV therapy in HIV-1 coinfected patients are very high, and premature treatment discontinuation due to serious adverse events ranges between 15% and 30%. Therefore, there is a need to improve treatment strategies in this specific group of coinfected patients in order to minimize side effects and treatment discontinuations and to maximize treatment success. Therapy is particularly recommended for coinfected patients with a high likelihood of achieving a SVR, i.e. patients infected with HCV genotype 2 or 3 and those infected with genotype 1 if the viral load is low (400 000–500 000 IU/mL) [23]. Insulin resistance and liver fibrosis tests are also recommended for patients with a low likelihood of achieving SVR.

The findings presented here confirm that HCV genotype 1 patients carrying an rs8099917 G allele have a low probability of treatment success; therefore, rs8099917 genotyping seems to be a valid pre-treatment approach for maximizing treatment success and minimizing HCV therapy-related toxicity. Coinfected patients with HCV genotype 4 may also benefit from rs8099917 genotyping. HCV genotype 4-infected patients carrying rs8099917 G alleles had similar rates of treatment failure as HCV genotype 1-infected patients. Nevertheless, the small number of HCV genotype 4-infected patients in our study prevented us from drawing conclusions about the predictive value of rs8099917 genotyping in this group of patients. Overall, our results are in agreement with those reported by Rallon et al. and Pineda et al. [19], [20], which have demonstrated a significant influence of the rs12979860 SNP, that is in linkage disequilibrium with rs8099917 [18], on the treatment response of HIV-1 patients coinfected with HCV genotypes 1 and 4. In the 160 patients of our study cohort, the rs12979860 SNP genotype was also highly associated with treatment success (P<0.0001, Chi-square test) (4.16; 2.2–8.21; P = 0.00003) (manuscript in preparation). However, it remains to be elucidated which of these two IL28B associated SNPs are more powerful to predict treatment success.

Similar to findings for HCV-monoinfected patients [18], the rate of treatment failure in patients infected with HCV genotype 3 was not affected by rs8099917 genotype, suggesting that the predictive value of genotyping this SNP is limited to patients infected with HCV genotypes that have low rates of treatment success. Genetic variation in IL28B and spontaneous clearance of HCV has also been described [18], [24]. In particular, the rs8099917 G allele increases the risk of progression to chronic HCV infection in both HCV-monoinfected and HCV/HIV-1 coinfected patients [18]. We found a significantly lower proportion of rs8099917 G alleles, 22%, in genotype 3-infected patients than would be expected given the prevalence of this allele (40% and 42%, in HCV genotype 1- and 4-infected patients, respectively). The prevalence of rs8099917 G alleles in HCV genotype 3-infected patients in our study cohort was within the ranges reported in an unrelated cohort of Swiss Caucasians (17%) [25] and in the Caucasian cohort of the Human Haplotype Map project (HapMap, www.hapmap.org) (15–19%); this suggests that the rs8099917 G allele may not affecting the rate of chronicity of HCV genotype 3. In contrast, the high prevalence of the rs8099917 G allele in HCV genotype 1- or 4-infected patients shows that the rs8099917 TT genotype may have a protective effect in terms of preventing the persistence of these two HCV genotypes. Since the rs8099917 G allele has been correlated with lower expression levels of IL28 genes [17], the different frequencies of the rs8099917 G allele in patients infected with different HCV genotypes may indicate that the innate immune system interacts differently with the different HCV genotypes. However, the viral factors involved in this interaction remain unknown. The IL28B, IL28A, and IL29 genes are closely related cytokine genes in chromosomal region 19q13 that encode proteins known as type III IFNs (IFN-λs) [26]. IFN-λ has been proposed as a possible treatment for hepatitis C [27], [28]. A phase 1b study involving 4 weeks of peg-IFN-λ plus RBV given to patients with chronic HCV infection showed that this combination had antiviral activity [27]. A comparison of the efficacy of IFN-λ against non-1 HCV genotypes may provide some clues about the interaction of this virus with the innate immune system.

An important question that remains to be answered is whether the rs8099917 genotype has higher treatment predictive value in HCV/HIV-1 coinfected patients than in HCV monoinfected individuals. Previous studies with HCV genotype 1-monoinfected patients found that the proportion of rs8099917 G allele carriers associated with treatment failure ranges from 50% to 87% [16], [17], [18]. In our cohort of patients coinfected with HIV-1 and HCV genotype 1, 88% of the rs8099917 G allele carriers failed therapy with peg-IFN-α/RBV. This percentage is within the high range of previous studies and suggests that IL28B genotyping may have a higher predictive value in coinfected patients. Further studies of cohorts with different ethnic profiles and treatment regimes will be necessary to determine whether there are differences between mono- and coinfected patients in regard to the predictive value of IL28B genotyping.

The recent development of compounds that directly inhibit HCV replication through interaction with viral proteins will increase the options for treating HCV infection. These compounds, which are now in phase 1, 2, and 3 trials, include reagents that target the HCV nonstructural (NS) 3 protease, the NS5A protein, and the RNA-dependent RNA-polymerase NS5B [29]. Data from phase 2 studies show that SVR rates improve significantly in patients receiving triple therapy consisting of an NS3 protease plus peg-IFN-α/RBV [30]. However, triple therapy has not been effective in all patients infected with HCV genotype 1 because of side effects, non-responsiveness to peg-IFN-α or RBV, or development of NS3 protease resistance. The rate of triple therapy treatment failure may be higher in HCV/HIV-1 coinfected patients. Most of the coinfected patients carrying IL28B risk alleles treated with this triple therapy may respond only to the NS3 protease inhibitor. Monotherapy with specific viral inhibitors poses a higher risk of selection of resistant variants and treatment failure [31]. Taken together, these results strongly suggest that more than one specific HCV inhibitor will be needed to successfully treat patients at high risk for non-response to peg-IFN-α/RBV treatment.

## Materials and Methods

### Patients

A total of 160 HCV/HIV-1 coinfected patients from our HIV clinic unit who had a standard course of treatment with peg-IFN-α/RBV with known virological response status at 24 weeks post-treatment were included in this study. Patient characteristics are shown in Table 1. Treatment success (i.e. achieved SVR) was defined as undetectable plasma HCV RNA using a sensitive RT-PCR assay 24 weeks post-treatment cessation. HCV genotype, a marker for hepatitis B virus (HBV) infection, HCV and HIV-1 viral load, CD4+ T cell count, and liver enzymes levels were determined using standard procedures. Severe fibrosis was considered in patients with a METAVIR score ≥F3. Written informed consent was obtained from each patient who participated in the study. Likewise, ethics approval was obtained from our Institutional Review Board (Hospital Universitari Germans Trias i Pujol).

### DNA collection and extraction

Blood was collected into EDTA tubes following standard procedures. Genomic DNA was extracted from peripheral blood mononuclear cells (PBMCs) using the QuickExtract DNA Extraction Protocol (EPICENTRE Biotechnologies). Briefly, 2×10^6^ cells were resuspended in 0.5 mL of QuickExtract Solution, incubated at 65°C for 10 min, incubated at 98°C for 2 min, and then stored at −70°C.

### rs8099917 SNP genotyping

SNP genotyping was performed by PCR amplification and direct PCR sequencing. The oligonucleotides used for PCR were rs8099917–128 (5′-GTGCATATGTTTTCTGAC-3′, sense) and rs8099917-556 (5′-GAGGCCCCTCACCCATGC-3′, antisense). The PCR amplification mixture contained 5 µL of PBMC genomic DNA solution, 10 pmol of each oligonucleotide, 200 µM deoxyribonucleoside triphosphates (dNTPs), 2 mM MgSO4, 1x high-fidelity PCR buffer (Invitrogen), and 0.25 U Platinum *Taq* DNA polymerase (Invitrogen) in a total reaction volume of 50 µL. Cycling parameters were one cycle of denaturation at 94°C for 2 min, 40 cycles of denaturation at 95°C for 30 s, annealing at 55°C for 30 s, and extension at 68°C for 30 s. Extension was followed by a 7-min incubation at 68°C. The resulting 430-nt PCR product was sequenced using two flanking PCR oligonucleotides, termed rs8099917–128 and rs8099917–556, with the Big Dye v3.1 kit and the 3100 DNA sequencing system (Applied Biosystems) as described previously [21]. Sequence alignment and editing was performed with the Sequencer version 4.1 (GeneCodes) software program. Hardy-Weinberg equilibrium was calculated using the Hardy-Weinberg Calculator software as implemented in http://www.tufts.edu.

### Statistical analysis

The association between the rs8099917 SNP and the response to peg-IFN-α/RBV treatment was assessed by a two-sided Chi-square test. The Mann-Whitney U test, unpaired t test, and two-sided Chi-square tests used to analyze baseline covariates were performed using GraphPad Prism version 4.00 for Windows (San Diego, CA, USA). Univariate and multivariate logistic regression analyses were used to determine the predictors of treatment success. We calculated the odds ratios and 95% confidence intervals. P values less than 0.05 were considered significant. Regression analyses were performed using the STATISTICA software version 9.1 (StatSoft Inc., Tulsa, OK, USA).
